# Let's talk about diet—Nutrition skills in medical education

**DOI:** 10.1111/medu.14770

**Published:** 2022-03-14

**Authors:** Allie Bradley, Jennifer Crowley, Harsh Bhoopatkar, Miriam Nakatsuji, Clare Wall

## 1 WHAT PROBLEM WAS ADDRESSED?

Doctors are recommended to support patients to improve their dietary behaviours to help reduce the prevalence of lifestyle chronic disease. The findings from a recent systematic review which investigated medical nutrition education throughout the world found that it was insufficiently incorporated into medical education, regardless of country, setting or year of medical education.^1^ Innovative and effective ways to increase students' nutrition knowledge, skills and confidence to counsel are required without increasing overcrowded curricula.

## 2 WHAT WAS TRIED?

A pilot intervention to improve Year‐4 medical students' knowledge and communication skills about nutrition and diet was developed and implemented by a multidisciplinary team from the University of Auckland, New Zealand. The pilot was included in the 1‐week General Practice Observed Patient Simulation (GPOPS) teaching block primarily focused on communication skills development in the context of simulated general practice consultations. This was considered an ideal place to introduce and practice nutrition‐related curriculum content and skills.

The education intervention included:

1. Online nutrition and Motivational Interviewing (MI) resources (MI guidelines and video demonstrating OARS(Open questions, Affirmations, Reflective listening and Summarising)
2. An introduction to the 5As (Ask, Assess, Advise, Agree, Assist) of Obesity Management
3. Healthy Conversation Skills (a patient‐centred approach to support life behaviour change) including a video to enhance healthy conversations skills
4. An MI tutorial to practice skills‐related to nutrition using role play with a peer
5. A simulation weight management/Pre‐Diabetes case with a standardised patient (actor) to practise MI interviewing and nutrition counselling skills followed by a debrief with a tutor

## 3 WHAT LESSONS WERE LEARNED?

On completion of each GPOPS teaching block, students were invited to complete a questionnaire to evaluate their perceptions of the resources provided and to canvas their opinions on the types of nutrition skills development that they would find useful. Additionally, students completed NUTCOMP, a reliable and validated questionnaire that assessed self‐perceived competence of primary health professionals in providing nutrition care to patients with lifestyle‐related chronic disease.

The MI resources provided to students in GPOPS were perceived most useful for increasing knowledge and confidence to counsel in nutrition. Similarly, observation of the patient simulation case increased students' perceived confidence to counsel patients. Opportunities for naturally incorporating and reinforcing nutrition skills suggested by the students included small group activities during pre‐clinical years involving problem‐based learning and role‐playing to strengthen communication skills. Students also suggested that consideration be given to the importance of interdisciplinary teaching and faculty collaboration to appropriately link, sequence and frame curricular content to create an integrated nutrition curriculum. Students' self‐perceived nutrition competence scores demonstrated positive attitudes towards nutrition, lack of confidence in nutrition knowledge and skills and moderate confidence to counsel in nutrition.

The pilot intervention aimed to integrate a modest amount of nutrition content focused on communication skills relating to diet and nutrition within the time constraints of the GPOPS teaching block. It complimented without compromising teaching of other important related aspects of lifestyle intervention.
